# Defense cells profile of cervical mucous during follicular and luteal phases of estrus cycle in river buffalo

**Published:** 2012

**Authors:** Esmail Ayen, Shapour Hasanzadeh, Saleh Tabatabaei

**Affiliations:** 1 *Department of Clinical Sciences, Faculty of Veterinary Medicine, University of Urmia, Urmia, Iran;*; 2 *Department of Basic Sciences, Faculty of Veterinary Medicine, University of Urmia, Urmia, Iran;*; 3 *Department of Animal Sciences, Faculty of Animal and Food Sciences, Ramin Agriculture and Natural Resources University, Ahwaz, Iran.*

**Keywords:** Buffalo, Cervical discharge, Defense cells, Estrous cycle

## Abstract

The aim of this study was to evaluate the defense cells changes of cervical mucous during follicular and luteal phases of estrus cycle in river buffalo. Reproductive organs of the adult and apparently healthy female buffaloes were collected from the slaughterhouse. By visual investigation of both the ovaries for presence of corpus luteum and growing follicles, the luteal and follicular phase of each buffalo was specified. Cervical discharge samples were collected by sterile swabs and then spread over the glass slides, dried and fixed with methanol. The specimens were undergone Giemsa staining. The percentage of lymphocytes, neutrophils, monocytes (macrophages), eosinophils and basophils in each case (for both the follicular and luteal phases) were obtained at 20 microscopic fields. The percentage of lymphocytes, neutrophils and basophils in luteal phase were higher than the follicular phase. The percentage of eosinophils in follicular phase was higher than the luteal phase. The percentage of monocytes (macrophages) in luteal and follicular phases was nearly equal. The statistical analysis showed that the differences of all cells between follicular and luteal phase were not significant (*P* > 0.05). The most defense cells in discharges of external os of cervix (both follicular and luteal phases) were neutrophils and lymphocytes.

## Introduction

While artificial insemination is used routinely on cow reproduction, this technique has not been used frequently for buffalo, partly because of poor signs of estrus. If there was a way for prediction of estrus stage, it would be possible to enhance the accuracy of estrus detection in buffalo. On reproductive tract discharge of cattle, the percentage of epithelial cells in the follicular phase was higher than luteal phase.^1^ The cervix acts as a sphincter-like fibrous organ and the main physiological function of that is to discharge a mucus secretion within the female reproductive tract. This secretion, stimulated by estrogen and produced by the simple columnar epithelial lining cells, includes mucous producing and ciliated cells. The tunica mucosa of the cervix is organized into primary and secondary folds to increase the surface area of this organ. An inner circular and outer longitudinal layer with smooth muscle and elastic fibers compose the tunica muscularis, and the cervix has a typical tunica serosa.^2^ Various types of epithelial cells are found in vaginal smears taken during the estrous cycle. Parabasal cells are the smallest. They are round cells with round nuclei, and have the highest nucleocytoplasmic ratio of any of the sloughed cells. Intermediate cells are larger than parabasal cells. Their nuclei are similar in size and shape to those of the latter. The corners of intermediate cells are rounded. Superficial intermediate cells (transitional cells) are bigger than intermediate cells and have angular edges. Their nuclei resemble those of parabasal and intermediate cells. Superficial cells are similar in size to superficial intermediate cells. Their edges are angular and may be folded. Their nuclei are pyknotic, faded, or lacking.^3^ Vaginal cytology provides a way of determining stages of the estrous cycle of the bitch or queen. On vaginal smear of more animals, the vast majority (90% or more) of cells during follicular phase are superficial cells. However during luteal phase, superficial cells decrease by a minimum of 20% and parabasal and intermediate cells which may have been absent or very sparse increase to more than 10% and sometimes frequently rise to more than 50%. There are no available reports regarding various types of epithelial cells in cervical smears during the estrus cycle of buffalo. The mechanisms which are preventing the opportunist microorganisms colonizing in the genital tract are, firstly, the physical barriers of the vulval sphincter and cervix and secondly, the natural defense mechanism of local tissues that are influenced significantly by the endocrine system.^1^ In most species including cattle, under estrogen dominance (follicular phase) the genital tract is more resistant to infection, whilst under progesterone dominance (luteal phase) it is more susceptible.^4^^,^^5^ During follicular phase of the estrus cycle there is a significant rise in estrogen level and a numerical change in the peripheral blood picture with a relative neutrophilia along with the migration of defense cells from the circulation to the reproductive tract which bring about active phagocytosis of microorganisms.^4^ The purpose of this study was to determine the cytological alternations of defense cells (lymphocytes, neutrophils, monocytes -macrophages-, eosinophils and basophils) of the cervical external os mucosal smears in river buffalo during follicular and luteal phases of estrus cycle.

## Materials and Methods

Reproductive tracts of 100 adult and apparently healthy female cyclic buffaloes (50 at follicular and 50 at luteal phase) were collected immediately after the slaughter in the Urmia abattoir. Tracts with different abnormalities, infections, pregnancy and recently calf delivery were eliminated. In each tract, by the precise inspections of both ovaries, the luteal and follicular phases were specified. The presence of developed or developing follicle with regressed corpus luteum were indicators for follicular phase, and the presence of developed or developing corpus luteum without developed follicle were indicators of luteal phase.^1^ Mucosal smears were collected using sterile swaps from external os of the cervix from each reproductive tract and then spread over the glass slides, then dried and fixed with methanol. The specimens were processed through routine Giemsa staining method.^6^ The percentage of lymphocytes, neutro-phils, monocytes (macrophages), eosinophils and basophils in all the cases (for both the follicular and luteal phases) were obtained at 20 microscopic fields.^7^ Microscopic observation was performed by light microscope using 1000× magnification. Results expressed as mean ± SE of percentages. Significance was assigned at *P *< 0.05. Data were analyzed using a statistical program (SPSS, version 14). Student *t*-test was carried out to find out the differences between mean percentage of each defense cell during two follicular and luteal phases.

## Results

The percentage of lymphocytes, neutrophils, monocytes (macrophages), eosinophils and basophils during follicular and luteal phases of estrus cycle are presented in Table 1. The percentage of lymphocytes, neutrophils and basophils in the luteal phase was higher than the follicular phase, whereas the percentage of eosinophils during follicular phase was higher than the luteal phase. The percentage of monocytes (macro-phages), in the luteal and follicular phases was nearly equal. For all defense cells, the differences of cell distributions in two phases were not significant (*P* > 0.05). The maximum percentage of different defense cells distribution during luteal and follicular phases belonged to neutrophils (18.83 ± 3.23 and 14.80 ± 3.14, respectively), and the minimum percentage belonged to basophils (0.03 ± 0.02 and 0.00 ± 0.00, respectively). 

**Fig 1:**
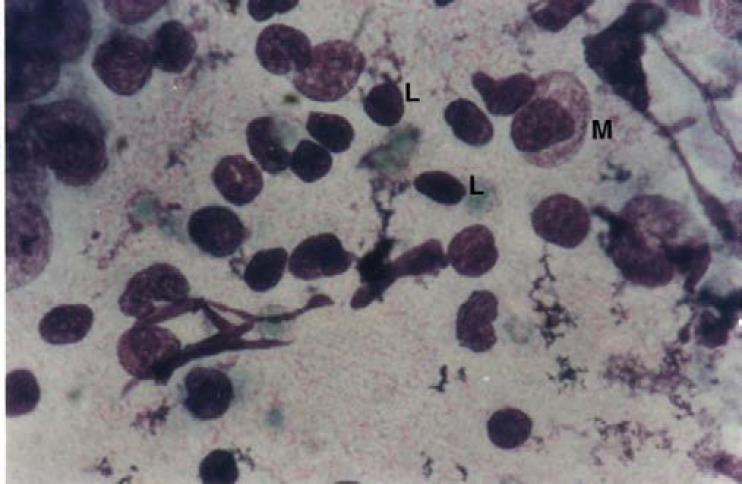
Cervical mucosal smears of buffalo during estrus cycle. Follicular phase: Monocyte (macrophage) (M) and Lymphocyte (L). Giemsa staining, 1000×

**Fig 2:**
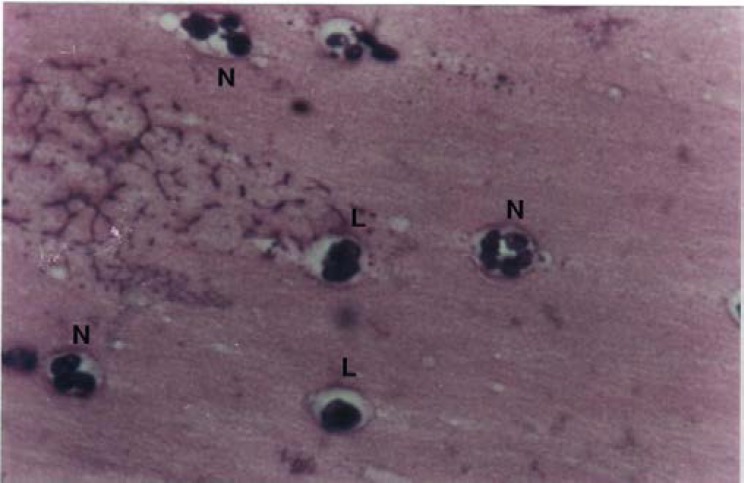
Cervical mucosal smears of buffalo during estrus cycle. Luteal phase: Lymphocyte (L) and neutrophil (N). Giemsa staining, 1000×

In Figs 1 and 2, some of the different defense cells can be observed at follicular and luteal phases in the mucosal smears of the cervical external os.

**Table 1:** Percentage of different defense cells of cervical mucosal smears in follicular and luteal phases of estrus cycle in buffalo

**Estrus phase**	**Lymphocytes (%)**	** Neutrophils (%)**	**Monocytes (%)**	**Eosinophils (%)**	**Basophils ** **(%)**
**Follicular**	13.32 ± 1.69	14.80 ± 3.14	0.72 ± 0.30	0.40 ± 0.21	0.00 ± 0.00
**Luteal**	15.03 ± 1.72	18.83 ± 3.23	0.75 ± 0.17	0.10 ± 0.04	0.03 ± 0.02

The differences of means in each column were not significant (*P* > 0.05).

## Discussion

The genital system of ruminants undergoes considerable changes during estrus cycle. These changes can be used in interpreting and diagnosing of different stages of estrus cycle. According to Ayen,^8^ Ayen *et al*.,^5^ Hasanzadeh *et al.*,^9^ and Miroud and Noakes^10^; vaginal mucosa of the ewe and cow undergo considerable histological changes during pregnancy and estrus cycle. Vaginal and cervical cytology may assist the diagnosis of the stage of the reproductive cycle in the dog and cat^11^^,^^12^ and this forms the basis for a pregnancy test in the ewe and sow.^13^ This study on river buffalo revealed that percentage of different defense cells between follicular and luteal phase were not significant (*P* > 0.05) and unlike to other species that have been investigated, in buffalo, the defense system of reproductive system is higher in all phases of estrus cycle. Faundez *et al*.^14^ indicated that percentage of polymorphonuclears on estrus to day 4 of estrus cycle in cattle is higher than other phases of estrus cycle. 

In Buffalo, because of the above defense system of reproduction tract on all phases of estrus cycle, the reproduction infection rate is lower than that in cattle. The reason for above defense system of buffalo reproductive system on all phases of estrus cycle is not clear. The maximum percentage of different defense cells distribution during luteal and follicular phases belonged to neutrophils (18.83 ± 3.23 and 14.80 ± 3.14, respectively), and the minimum percentage belonged to basophils (0.03 ± 0.02 and 0.00 ± 0.00, respectively). The same results have been reported by Ahmadi *et al*. in cow.^4^ In this study, during both the phases (follicular and luteal), percentage of neutrophils in reproductive tract was higher than lymphocytes that was similar to results of Ahmadi *et al*. in cow.^4^ Reagan *et al*. indicated that in circulating blood of buffaloes, percentage of lymphocytes is higher than neutrophils.^15^ This phenomenon has been explained by Hussain, according to him; the immediate defense cells of the genital tract able to show a response to an infection was neutrophils arriving to the area earlier than lymphocytes.^16^ This study demonstrated that the majority of defense cells in discharges of external os of cervix in both follicular and luteal phases were neutrophils and lymphocytes.

In conclusion, we can state that unlike to cow, defense cell feature of cervix could not be applicable for evaluation and determination of phases of estrus cycle in river buffaloes.

## References

[1] Arthur GH, Noakes DE, Pearson H (1996). Veterinary Reproduction and obstetrics.

[2] Schatten H, Constantinescu GM (2007). Comparative Repro-ductive biology.

[3] Bacha WJ, Bacha LM (2000). Color atlas of Veterinary histology.

[4] Ahmadi MR, Nazifi S, Gheisari HR (2000). Cytology changes in heifers, cervical mucosa at different phases of the estrus cycle.

[5] Ayen E, Hasanzadeh S, Abdollahvand M (2002). Alternations in mucosa membrance of vagina during different stages of pregnancy, estrus and di-estrus in cow. J Vet Med Univ Tehran.

[6] Gretchen LH (1979). Animal Tissue Techniques.

[7] Dellman HD, Eurell J (1998). Textbook of Veterinary Histology.

[8] Ayen E ( 1996). Factors involved in the cause and patho-genesis of cervico-vaginal prolapse in the ewe PhD Thesis.

[9] Hasanzadeh S, Ayen E, Khalilzadeh O (2004). Histological and histomorphometric alterations in the cranial and caudal vaginal mucous membrane of HF cow during follicular and luteal phases of estrus cycle. Indian J Anim Sci.

[10] Miroud K, Noakes DE (1991). Histological changes in the vaginal mucosa of the cow during the estrus cycle, after ovary-ectomy and following exogenous estradiol benzoate and progesterone treatment. British Vet J.

[11] Simpson G, England G, Harvey M ( 1998). BSAVA Manual of Small Animal Reproduction and Neonatology.

[12] Watts JR, Wright PJ, Lee CS (1998). Endometrial cytology of the normal bitch throughout the reproductive cycle. J Small Anim Prac.

[13] Robertson HA (1980). Diagnosis of pregnancy in the ewe at midgestation. Anim Reprod Sci.

[14] Faundez R, Duszewska AM, Klucinski W ( 1994). Occurrence of leukocytes and epithelial cells in the lumen of the reproductive tract during the ovarian cycle.

[15] Reagan WJ, Sanders TG, Denicola DB (1998). Veterinary hematology, Atlas of common domestic species.

[16] Hussain AM (1989). Defense cells of reproductive system. J Vet Med.

